# A State of Independents: Rationalizing the High *Z*ˈ Crystal Structures of Shikimate Esters

**DOI:** 10.1021/acs.cgd.3c01383

**Published:** 2024-01-16

**Authors:** Ronnie Ragbirsingh, Michael J. Hall, Michael R. Probert, Paul G. Waddell

**Affiliations:** Chemistry, School of Natural and Environmental Sciences, Newcastle University, Bedson Building, Newcastle upon Tyne NE1 7RU, U.K.

## Abstract

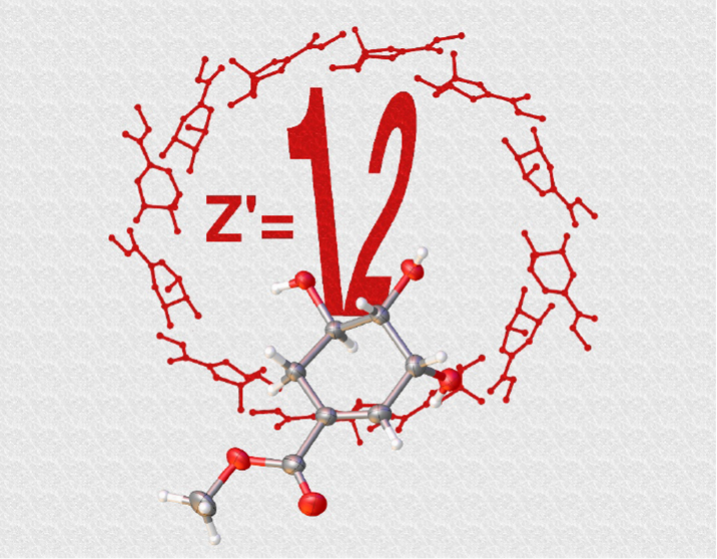

*Z*ˈ is a parameter used to denote the number of symmetry-independent molecules in the asymmetric unit of a crystal structure. High *Z*ˈ (>1) crystal structures are relatively uncommon and are thought to arise through competition between intermolecular interactions of similar strength. As such high *Z*ˈ crystal structures are challenging to predict and new examples are valuable in improving understanding in the field. Herein, we report the X-ray crystal structures of a series of shikimate esters, the asymmetric units of which exhibit high *Z*ˈ values. Of special interest is the crystal structure of methyl shikimate, the asymmetric unit of which comprises 12 independent molecules; *Z*ˈ = 12. This uncommonly large *Z*ˈ value arises through a combination of factors, including the intrinsic homochirality of the molecule, the conformational inflexibility of the cyclohexene ring, the presence of multiple hydrogen bonding motifs, and both the *cis*- and *trans*-conformers of the ester moiety. Comparison of the X-ray crystal structures of shikimic acid, methyl shikimate, ethyl shikimate, and *iso*-propyl shikimate suggests that instances of high *Z*ˈ in this series correlate with specific hydrogen bonding motifs influenced by the steric bulk of the ester. The results of this study provide important insights into factors that influence the formation of organic crystal structures where the value of *Z*ˈ is greater than 1.

## Abstract 2

A series of shikimate ester crystal structures was determined and their propensity to form asymmetric units of more than one molecule was rationalized through an analysis of the packing.

## Introduction

The phenomenon of crystal structures with asymmetric units that comprise multiple chemically identical yet crystallographically independent molecules is one that has been known to and has intrigued the field of crystallography for much of its history.^1−3^ More recently, structures of this kind have been studied to aid developments in crystal structure prediction and the understanding of polymorphism.^4,5^ In homomolecular crystals, the number of molecules in the asymmetric unit is defined as *Z*ˈ. Typically, where *Z*ˈ >1 the structure is referred to as having a high *Z*ˈ. High *Z*ˈ crystal structures are still relatively uncommon accounting for only 10% of all structures in the June 2023 version (5.44) of the Cambridge Structural Database (CSD). Due to the complexity of high *Z*ˈ crystal structures, more recently they have become important case studies in the development of crystal structure prediction tools and in the understanding of polymorphism.^4,5^

A large number of factors that influence the formation of crystal structures with high *Z*ˈ have been identified related to the properties of the molecule (e.g., chirality, shape, and conformational flexibility^6−8^), the packing within the crystal (e.g., pseudosymmetry, modulation, and intermolecular interactions^9−12^), and even the method used to produce the crystals.^13^ In the majority of cases where *Z*ˈ >1, these factors contribute to competition between intermolecular interactions of similar strength within the structure. Though the phenomenon has been reported and analyzed extensively, as detailed by the Steeds in their definitive 2015 review of the topic,^14^ predicting occurrences of high *Z*ˈ still poses a great degree of difficulty^4^ and hence there is much to be learned from new case studies of structures that possess this unusual property. In addition, most reported high *Z*ˈ crystal structures are chemically isolated examples, making it hard to identify the key contributions of the intramolecular forces involved in the crystal structure.

This work concerns structures related to shikimic acid, a natural product first isolated by Eykman in 1885 from the fruit of the Japanese star anise tree *Illicium religiosum*,^15^ with structure elucidation carried out by Fisher et al. some 50 years later.^16^ The fruits of plants of the genus Illicium can be up to 24% shikimic acid by dry weight, including the spice star anise from*Illicium verum*.^17^ Shikimic acid is an important intermediate in the shikimate pathway, found in plants and microorganisms, for the biosynthesis of folates, alkaloids, and aromatic amino acids (phenylalanine, tyrosine, and tryptophan). Shikimic acid is also a starting material in the industrial synthesis of the antiviral oseltamivir, known commercially as Tamiflu, a drug which is used to combat the H5N1 influenza virus.^18^

During our work on the synthesis of a biologically active natural product, shikimic acid was esterified to give the methyl shikimate ester (**1**).^19^ During the course of the characterization of **1**, single crystal X-ray crystallographic analysis revealed that this compound crystallizes with an unusually high *Z*ˈ value (*Z*ˈ = 12). To date, there are only 86 crystal structures in the CSD where *Z*ˈ is greater than or equal to 12 and hence we decided that a molecule exhibiting such a large *Z*ˈ value constituted a rarity worthy of further investigation. This serendipitous discovery also opened up a new avenue of investigation into the potential origins of the high *Z*ˈ phenomenon, as it would be straightforward for us to synthesize a range of alkyl shikimate esters and to study the crystal structures of this series. As far as we are aware, a systematic study of this kind aiming to produce a series of related compounds with a tendency to produce high *Z*ˈ crystal structures would be unique.^20^

Given the links between instances of high *Z*ˈ in crystal structures and our understanding of polymorphism, any insights gleaned from this study will be of significant interest in the field of crystal engineering.

**Scheme 1:**
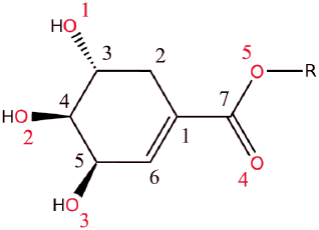
Common Fragment of the Shikimate Esters with Atomic Numbering Scheme (R = Me (**1**), Et (**2**), *i*-Pr (**3**))

To this end, the ethyl (**2**) and *iso*-propyl (**3**) esters of shikimic acid were also synthesized and their crystal structures, and that of the parent shikimic acid (**5**), were determined using single crystal X-ray crystallography. Within this group of compounds, only the R group is varied (Scheme 1) to provide a closely related and self-consistent set of molecules to facilitate the study. Analysis of hydrogen bonding, molecular conformation, and packing environments across these three crystals structures was undertaken to rationalize the instances where *Z*ˈ >1.

## Experimental Section

Shikimic acid was purchased from Fluorchem and was used without further purification. Methyl (**1**), ethyl (**2**), and *iso*-propyl (**3**) shikimate esters were prepared by literature methods.^21^ Further details of the synthesis and characterization of all compounds prepared for this article are available in the ESI. Crystals suitable for analysis by single crystal X-ray crystallography were grown by slow evaporation of the solvent from a solution of the compound in dichloromethane (**1**) or chloroform (**2** and **3**).

Crystals of a 2:1 cocrystal of shikimic acid and the *iso*-propyl shikimate ester (**4**) were isolated from the attempted crystallization of an impure sample of *iso*-propyl shikimate ester, in which the starting material was still present. Crystals of shikimic acid (**5**) were grown by slow evaporation of the solvent from a solution of the compound in methanol.

Crystal structure data for **1** were collected at 100 K on beamline I19^22,23^ at Diamond Light Source using synchrotron radiation (λ = 0.68890 Å) and the data processed using the software APEX3.^24^

Crystal structure data for **2**–**4** were collected at 150 K on an Xcalibur, Atlas, Gemini ultra diffractometer (Rigaku Oxford Diffraction) equipped with a sealed tube X-ray source (λ_CuKα_ = 1.54184 Å) and an Oxford CryostreamPlus open-flow N_2_ cooling device. For **2**, **3** and **5** intensities were corrected for absorption using a multifaceted crystal model created by indexing the faces of the crystal for which data were collected.^25^ For **4** the intensities were corrected for absorption empirically using spherical harmonics. Cell refinement, data collection and data reduction were undertaken via the software CrysAlisPro.^26^

All structures were solved using XT^27^ and refined by XL^28^ using the Olex2 interface.^29^ All non-hydrogen atoms were refined anisotropically, and hydrogen atoms were positioned with idealized geometry, with the exception of those bound to oxygen, the positions of which were located using peaks in the Fourier difference map. The displacement parameters of the hydrogen atoms were constrained using a riding model with *U*_H_ set to be an appropriate multiple of the *U*_eq_ value of the parent atom. Further details of the refinement and treatment of any disorder, along with figures for the asymmetric unit and, where appropriate, packing motifs for each structure, are included in the ESI and in the CIF files deposited in the CSD.

## Results and Discussion

Even though this discussion mainly focuses on the pronounced differences between the shikimate ester structures, it is worth highlighting the many things they all have in common. All of the crystal structures determined as part of this study crystallized in one of the Sohncke space groups, as is expected in the case of enantiomerically pure, chiral compounds. The structures of the homomolecular crystals and cocrystals of shikimate esters all form two-dimensional hydrogen bonding networks parallel to the crystallographic (001) plane, though the complexity and connectivity of these networks varies between structures. Hydrogen bonding ring motifs with the graph set R^2^_2_(10) that form between two molecules are also common to all shikimate ester structures.^30^ The homochiral nature of these shikimate esters precludes the two molecules involved in the formation of this motif being related by inversion symmetry; however, they are related by either a 2-fold rotation or pseudorotation.

In all cases, the cyclohexene ring of the shikimate ester adopts the energetically favorable half-chair conformation^31^ and hence there is very little conformational variation of this ring between the molecules of the various structures. Considering only the homomolecular shikimate ester structures, *Z*ˈ decreases in the order **1** > **2** > **3** with values of 12, 2, and 1, respectively. Given the rigidity of these molecules with respect to the cyclohexene ring, it might be expected that more atoms in the alkyl group of the ester moiety would allow for greater conformational flexibility and a greater probability of crystallizing with a higher *Z*ˈ^6^ but the opposite is observed to be the case. For **1**–**3**, *Z*ˈ is larger the smaller the ester group, and this can be rationalized by the decrease in steric bulk allowing the ester to more easily adopt different conformations within the same volume without forming unfavorable intermolecular interactions. This is highlighted by the case of **1**, where it is the presence of both *cis*- and *trans*-conformers of the ester group relative to the alkene double bond of the cyclohexene ring that influences the formation of the very high *Z*ˈ structure.

**Figure 1:**
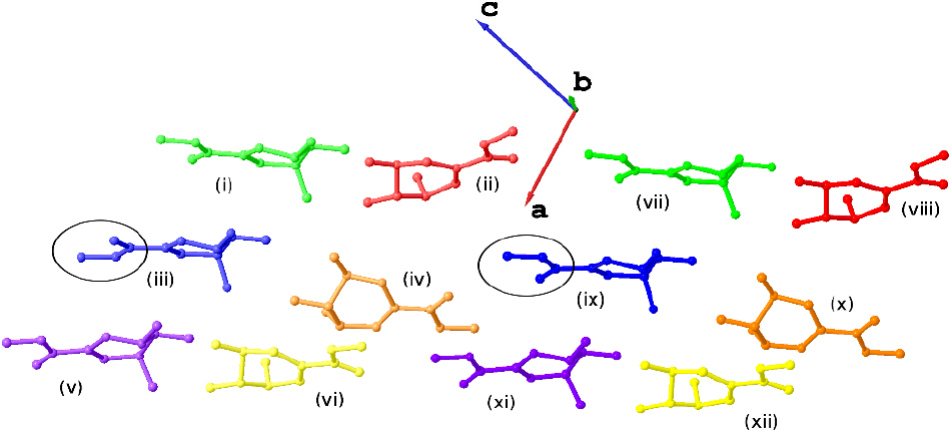
Asymmetric unit of **1** viewed approximately along the [010] direction. Each independent molecule has been assigned a label i-xii. i-vi (left) and vii-xii (right) comprise the two sets of six molecules related by an approximate translation. Pairs of atoms related by this approximate translation have been rendered in similar colors, and the conformational variation between iii and ix has been highlighted. Hydrogen atoms have been omitted for the sake of clarity.

The structure of **1**, where R = Me and the ester group has the lowest steric bulk of the shikimate esters studied, is by far the most unusual as its asymmetric unit is observed to comprise 12 crystallographically independent molecules (*Z*ˈ = 12). These will be referred to as i-xii, as per Figure 1. Within the asymmetric unit there are a number of relationships between the molecules, which need to be addressed if we are to understand the occurrence of high *Z*ˈ in this case.

**Scheme 2:**
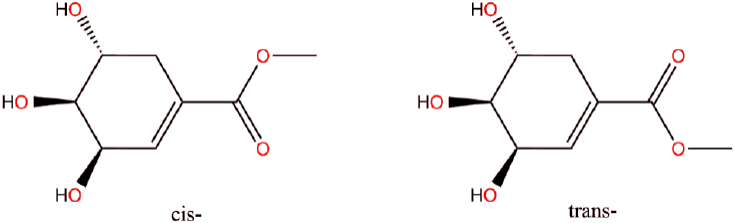
*cis*- and *trans*-Conformers of **1**

Within the asymmetric unit, the *cis*- and *trans*-conformations (Scheme 2) are observed in a 3:1 ratio to give 9 *cis*- and 3 *trans*-conformers. Given that this compound crystallizes in the space group P2_1_, in which there are only two general positions, the odd numbers of each conformer present preclude any further symmetry within the asymmetric unit and can thus be identified as one of the contributing factors to the high *Z*ˈ value. The very presence of the two conformers, which cannot be related by any crystallographic symmetry operation, means that the asymmetric unit must necessarily have *Z*ˈ >1 to accommodate both.

The effect of the presence of both conformers becomes clear when viewing the asymmetric unit down the crystallographic [010] direction (Figure 1). In this orientation, it can be seen that it comprises two very similar sets of six molecules. Each set of six is hydrogen-bonded together to generate coplanar layers of hydrogen-bonded molecules parallel to (001). A cursory glance at this view of the asymmetric unit may lead one to conclude that translating one set of six along the [101] direction may generate the second, but on closer inspection, this is not the case. Although five of the molecules would superimpose almost directly upon performing this operation, one of them (either iii or ix) would need to rearrange from *trans* to *cis* or vice versa to do the same and hence the two sets are not equivalent. This change in conformation implies that even though the other five pairs of molecules may be very similar in terms of their conformation and relationship to the other molecules in the set that remain the same upon translation, the exact packing environment for the molecules of each pair is different as a knock-on effect of the molecule whose conformation does change upon translation. The result of this is the 12 different packing environments for the 12 symmetry independent molecules that comprise the asymmetric unit. This feature can be characterized as an ordered fault in which the pseudo translation along [101] is broken by the changing conformation. Instances of this phenomenon in high *Z*ˈ structures are relatively rare.^20^

If we continue to consider the asymmetric unit as two groups of six molecules, then we can see that the hydrogen bonding network that links each set of six is almost identical. Within each set, we see two dimers that form the R^2^_2_(10) ring motif through interactions between the hydroxyl groups containing O1 and O2 on each molecule. These dimers are linked together by another pair of molecules, themselves connected by a single hydrogen bond, to form an R^6^_6_(24)[R^4^_4_(14)R^4^_4_(14)] motif across the whole set of six. The molecules that form the R^2^_2_(10) dimers are related by approximate 2-fold rotation symmetry (if the hydrogen atoms are ignored). Motifs such as this are common to **1**–**3** and this would suggest that the formation of the R^2^_2_(10) ring is favorable in these compounds. Within the asymmetric unit, there are two further pairs that do not exhibit this motif. This is potentially an example of “frustration”, where the molecules are unable to form the preferred dimer motif to prioritize close packing of the other dimer-forming molecules. This might be identifiable as another factor contributing to high *Z*ˈ in this structure.

While analyzing the hydrogen bonding in **1**, it is also worth highlighting that there appear to be three distinct hydrogen bonding environments within the asymmetric unit with four molecules in each. One may expect more given the 12 independent molecules, but this is a function of the way in which the hydrogen bonding network is formed. As the structure forms two-dimensional layers of hydrogen-bonded molecules, the molecules are arranged in a head-to-head and tail-to-tail fashion so that the three hydroxyl groups at one end of each molecule interact with those of adjacent molecules to form the hydrogen bonding network and the methyl groups of one layer are orientated toward those of another to form the boundaries between these layers. As the ester group, which is the only moiety in which any real conformational change is observed, does not interact with the hydroxyl groups, and the hydrogen bonding network is unaffected by this change.

What is noteworthy about the hydrogen bonding environments is that each hydroxyl group acts as a classical hydrogen bond donor (i.e., where the donor is a proton bonded to an electronegative heteroatom and the acceptor is an electronegative heteroatom), but the number of classical hydrogen bond acceptors in each environment varies. More specifically, the hydroxyl groups containing O1 and O2 act as both an acceptor and a donor in each of the independent molecules, but while the hydroxyl group containing O3 always acts as a donor, the number of classical hydrogen bonds it accepts varies from two to none.

**Figure 2:**
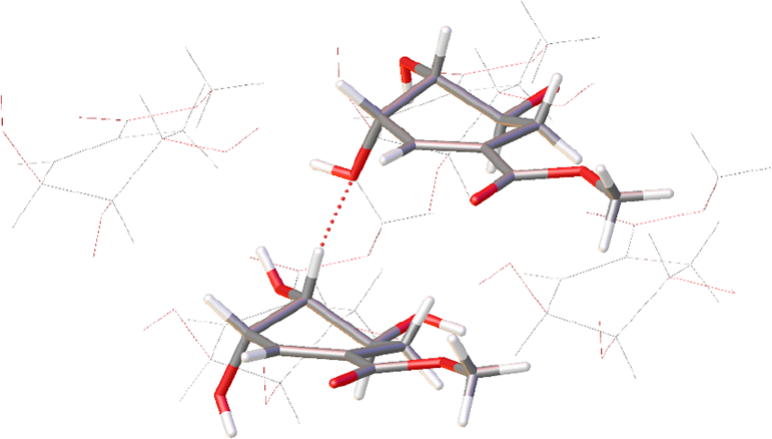
Hydrogen bonding environment about ii in the structure of **1** highlighting the weak C–H···O hydrogen bond.

The two constituent molecules of each R^2^_2_(10) dimer represent two of the three hydrogen bonding environments. Here, the difference between the two environments is that in one (populated by i, vi, vii, and xii), the hydroxyl group containing O3 accepts two hydrogen bonds to neighboring molecules, while in the second (populated by ii, v, viii, and xi), the same group accepts no classical hydrogen bonds. In this second case, O3 is the acceptor for a so-called “weak” hydrogen bond involving a C–H donor on a neighboring molecule (Figure 2). For ii, v, and viii, this donor is the proton on C4 where the H···O3 distance ranges from 2.40(1) to 2.54(1) Å, whereas for xi, the donor proton is bonded to C2 with an equivalent distance of 2.50(1) Å. The formation of this weak interaction in preference to another classical hydrogen bond is likely another example of frustration, where the close packing of the dimer units precludes a more favorable interaction. The discrepancy in the packing environment as to which proton acts as the hydrogen bond donor in this interaction for just one of the four molecules in which this interaction is observed is further testimony to the large *Z*ˈ value for this structure.

**Figure 3:**
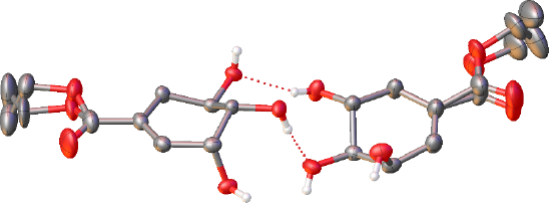
Asymmetric unit of **2** showing disorder of the ethyl ester groups. Displacement ellipsoids have been rendered at the 50% probability level and hydrogen atoms bound to carbon have been omitted for clarity.

By way of contrast to the structure of **1**, in the structure of **2** it appears that there is no frustration between the formation of the dimeric unit and the close packing of these dimers. As in **1**, molecules of **2**, the ethyl ester, exhibit the same hydrogen-bonded R^2^_2_(10) dimer motif involving the hydroxyl groups containing O1 and O2 between the two crystallographically independent molecules that comprise the asymmetric unit (*Z*ˈ = 2). Similar to the dimers in **1**, the two molecules are related by pseudosymmetry; an approximate 2-fold rotation about the [010] axis. In this instance, the symmetry is broken by the positions of the hydrogen atoms of the hydroxyl groups, and the disorder manifests in the ethyl ester groups (Figure 3). As each neighboring dimer is generated via the space group symmetry or crystallographic translation, it is simply this disorder that leads to multiple independent molecules in this structure. As a consequence, only the *cis*-conformation of the molecule is present in **2** precluding the formation of the larger asymmetric unit required to accommodate both conformers.

Bucking this trend of shikimate ester structures where *Z*ˈ >1 is the third homomolecular shikimate ester structure determined as part of this study: the *iso*-propyl ester, **3**. In this case the asymmetric unit comprises just one molecule (*Z*ˈ = 1). Ordinarily, this would be an unremarkable observation; however, in this case, there is a fundamental difference between the structure of **3** and those of the other homomolecular esters in this study, which results in a more symmetrical relationship between molecules in the crystal.

**Figure 4:**
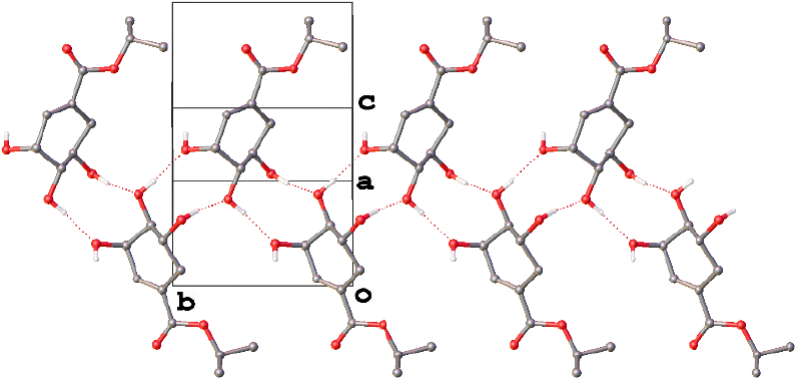
A view of the chain of molecules linked by hydrogen-bonded rings formed along [010] in the structure of **3**. Hydrogen atoms not directly involved in hydrogen bonding have been omitted for clarity.

The structure of **3** may appear superficially similar to those of **1** and **2** and one of these similarities is the formation of the R^2^_2_(10) hydrogen bonding motif between molecules. The difference in this instance is that where in **1** and **2** this motif involves the hydroxyl groups containing O1 and O2 and the molecules are related by approximate rotation symmetry, in **3** the R^2^_2_(10) ring forms as the hydroxyl groups containing O2 and O3 on one molecule act as hydrogen bond donors to the O1 and O2 atoms of a neighboring molecule. The involvement of all three hydroxyl groups in the formation of this motif generates a herringbone chain of R^2^_2_(10) rings along the [010] direction with each molecule related to the next by the symmetry of the 2_1_ screw axis (Figure 4). It is the translational element of this symmetry that is lacking between R^2^_2_(10) linked molecules in **1** and **2** and it is this that allows molecules of **3** to pack in such a way that each molecule is crystallographically equivalent.

The formation of this motif is likely the result of the molecules orienting themselves to avoid unfavorable close contacts between the ester groups occurring at the boundaries between the hydrogen-bonded layers. These interactions can be seen as increasingly unfavorable as the steric bulk of the ester group increases. In **1**, where the ester group is smallest, there is little to preclude the interaction between these groups that result from the direct head-to-head orientation of the molecules between layers regardless of whether the molecule is in the *cis*- or *trans*-conformation. In **2**, increasing the size of the group from methyl ester to ethyl ester results in the presence of only the *cis*-conformer and disorder of the ethyl groups, as these moieties begin to interact more strongly. When the ester increases in size further to the *iso*-propyl ester of **3** the direct head-to-head orientation is no longer feasible, and hence, the packing of the molecules in this direction changes drastically to accommodate the increased steric bulk.

Though it appears to have little bearing on *Z*ˈ, it is also worth noting that there is also a pronounced twist between the ester and cyclohexene moieties in **3** where barely any deviation from planarity is observed in molecules of **1** and **2**. This is best illustrated by the C6–C1–C7–O4 torsion angle (or C6–C1–C7–O5 in the case of the *trans*-conformers of **1**). In **3** this angle is 28.9(8)° whereas this angle averages ca. 7° and barely exceeds 12° for all of the symmetry-independent molecules in **1** and **2** combined.

Having considered the structures of the three homomolecular shikimate esters and rationalized the variation in the value of *Z*ˈ observed for each, it is tempting to conclude that the structures that exhibit high *Z*ˈ do so due to a series of coincidental factors specific to the individual structure and not due to some inherent property of shikimate esters as a whole. For the esters that do exhibit high *Z*ˈ, the cause of this property appears to be different for each even though there are some fundamental similarities between the structures. At this stage it would potentially be useful to analyze known structures of molecules containing the shikimate moiety; however, **1**–**3** represent the only structures of shikimate esters yet determined and there are no crystal structures containing shikimic acid in the Cambridge Structure Database (CSD). In fact, only one structure containing the shikimate moiety, the dihydrate form of the salt sodium shikimate (CSD REFCODE: VUXROV), has so far been made available in the CSD.^35^ Though it is not pertinent to draw conclusions from such a small data set, somewhat encouragingly, as the asymmetric unit of this structure comprises two equiv of the salt and four water molecules, the value of *Z*ˈ is 2. Though it may not be that the direct cause is the same, it does seem to lend credence to the idea that shikimates are more likely to crystallize with *Z*ˈ> 1 than not.

A further indication of this is seen in the structure of **4**, a 2:1 cocrystal of shikimic acid and the *iso*-propyl shikimate ester, reported here as the first structure to contain the shikimic acid molecule. Though formally the structure has a *Z*ˈ value of 1 as the 2:1 ratio cannot be divided to give a smaller integer ratio, in this instance, as **4** is a cocrystal, the structure can be compared to those of **1**–**3** in terms of the value of a parameter related to *Z*ˈ, namely, *Z*ˈˈ. Zˈˈ is the number of independent chemical species in the asymmetric unit of a crystal structure and hence for **4***Z*ˈˈ = 3. By this definition, *Z*ˈˈ = Zˈ for homomolecular crystals such as **1**–**3**. Instances of high *Z*ˈˈ are not as well documented as those of high *Z*ˈ but can provide similar insights into the formation of cocrystal structures.^32−34^

The high *Z*ˈˈ value, the fact that there are two crystallographically independent molecules of shikimic acid in the asymmetric unit, and the structural similarity between the two chemical components of the cocrystal make the structure of **4** directly comparable to those of **1**–**3**. In specific terms, even though the presence of the two independent shikimic acid molecules is clearly a consequence of the uneven ratio of the constituent chemical species, it is the intermolecular interactions between the molecules in the cocrystal that can provide some insight into why high *Z*ˈ values are likely in shikimates.

Regarding the shikimic acid molecule, the most obvious difference between it and the shikimate esters discussed thus far is the presence of the carboxylic acid group. This equips shikimic acid with an extra hydrogen bond donor relative to the esters and a greater range of possibilities, when it comes to potential hydrogen bonding motifs within the crystal. One might imagine that, as is sometimes observed, a dimer forming between the two carboxylic acid groups with an R^2^_2_(8) motif may be a favorable configuration. Without any knowledge of the atomic positions, the 2:1 ratio between shikimic acid and shikimate ester might seem to suggest this; it is easy to imagine what is essentially a 1:1 cocrystal of dimer and ester.

**Figure 5:**
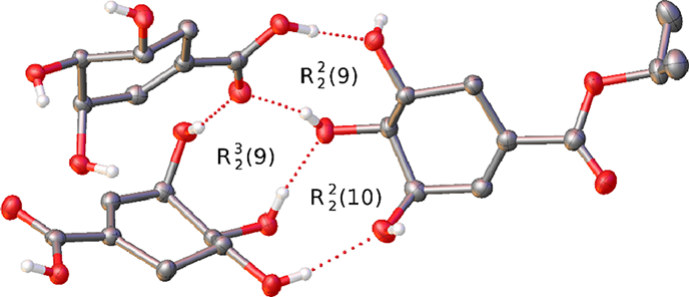
Asymmetric unit of **4** showing the fused hydrogen-bonded ring system. Displacement ellipsoids have been rendered at the 50% probability level and hydrogen atoms bound to carbon have been omitted for clarity.

In the structure of **4**, the proposed R^2^_2_(8) motif formed of two carboxylic acid groups is not observed. Instead, a variety of other hydrogen bonding ring motifs form among the three molecules in the asymmetric unit (Figure 5). The ubiquitous R^2^_2_(10) ring motif observed in **1**-**3** can be seen between the propyl ester molecule and one of the acid molecules but, as was observed in the structure of **1**, there are also molecules in the structure that do not form this motif. The presence of the carboxylic acid groups in the asymmetric unit allows for the formation of a R^2^_2_(9) ring between the acid group and two of the hydroxyl groups on a neighboring molecule. The acid group of one independent shikimic acid molecule forms this ring with the other acid molecule which in turn forms the same motif with the hydroxyl groups of the ester. Together the R^2^_2_(10) ring and the R^2^_2_(9) ring between the acid and ester molecules form a fused ring system and allows for the close approach of the hydroxyl group of an acid molecule that is not involved in either the aforementioned rings to form a third hydrogen-bonded ring between all three molecules with the graph set motif R^3^_2_(9), a motif that can also be observed elsewhere in the structure linking three adjacent shikimic acid molecules.

That the acid group allows the more planar end of the molecule to be involved in hydrogen bonding appears to be the reason for the formation of the cocrystal and the 2:1 acid–ester ratio. In the structures of the esters, hydrogen bonds could only be formed at one end of the molecule, the more awkwardly shaped end, in terms of packing. It seems that it is the fact that this asymmetric moiety must necessarily form hydrogen bonds to other molecules and that adjacent molecules held together by hydrogen bonds are unlikely to be related by symmetry due to their awkward shape that leads to the tendency of these molecules with this moiety to crystallize with high *Z*ˈ and *Z*ˈˈ values.

It is also worth noting that the twisting of the cyclohexene ring and the *iso*-propyl ester group observed in the structure of **3** is also apparent in **4**, where a similar C6–C1–C7–O4 torsion angle of 29.7(8)° is observed. In contrast, the equivalent torsion angles in the shikimic acid molecules in **4** are much smaller and comparable to those of **1** and **2**.

Analysis of the structures of **1**–**4** raises a fairly obvious question: would the crystal structure of shikimic acid itself exhibit a high *Z*ˈ value? Would the absence of the alkyl group, which acts to prevent the hydrogen bonding motifs from propagating in certain directions in **1**–**4**, allow shikimic acid to pack in a more symmetrical manner or would it form a structure more similar to that of the cocrystal **4**, which contains molecules of shikimic acid and has a *Z*ˈˈ of 3? To this end, and due to the glaring absence of the structure of such an important molecule from the literature, crystals of shikimic acid were grown, and the structure was determined by X-ray crystallography.

Shikimic acid (**5**) crystallizes with one crystallographically independent molecule in the asymmetric unit (*Z*ˈ = 1). Analysis of the packing in this structure, specifically the hydrogen bonding network that forms between the molecules in the solid state, gives a clear indication as to why this is the case and why it is not for many of the alkyl shikimate esters. One striking observation is that the R^2^_2_(10) ring formed in each of the shikimate ester containing structures is absent. Instead, the R^2^_2_(9) ring between the acid group and two of the hydroxyl groups on a neighboring molecule observed in the structure of **4** is formed. This suggests that where possible, the R^2^_2_(9) motif forms in preference to the R^2^_2_(10) ring.

**Figure 6:**
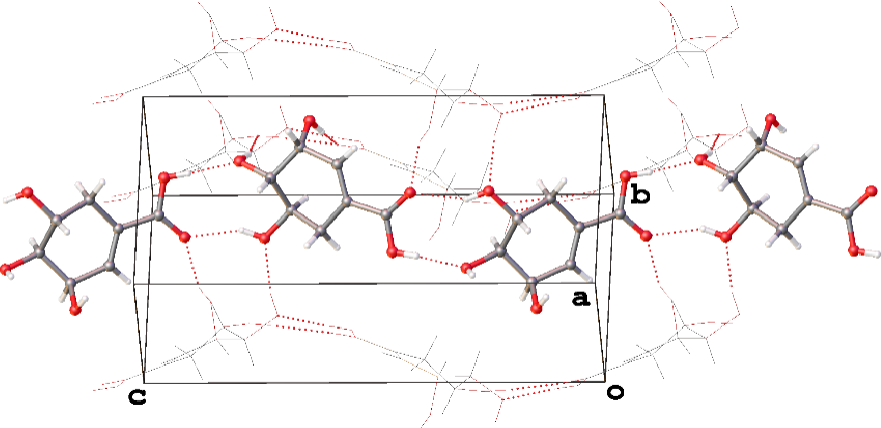
A view of the chain of hydrogen-bonded shikimic acid molecules along the [001] direction within the 3D hydrogen bonding network of structure of **5**. Hydrogen atoms not involved in hydrogen bonding have been omitted for clarity.

The molecules linked by these R^2^_2_(9) rings form chains of molecules hydrogen bonded in this fashion along the crystallographic [001] direction (Figure 6). In turn, these chains are linked by R^3^_2_(9) hydrogen-bonded rings involving three adjacent molecules, a motif which was again a feature of the structure of **4**, to create a three-dimensional hydrogen-bonded network. This is in stark contrast to the exclusively two-dimensional hydrogen bonding networks of **1**–**4**.

Given the occurrence of identical R^2^_2_(9) and R^3^_2_(9) motifs in both structures, **5** can be said to closely resemble **4**, which is not surprising, as they share a constituent molecule. The presence of the alkyl group in **4** is what appears to precipitate the differences in packing between these structures, and in a broader sense this can be seen as one of the main causes of the frustration between intermolecular symmetry and close packing in all the shikimate ester structures.

The chain motif apparent in the structure of **5** is a feature that it shares with the structure of **3**. In both cases, this chain of molecules propagates along a 2_1_ screw axis so that each molecule in the chain is related to its neighbor by this symmetry. Both structures also have only one molecule in the asymmetric unit. From this observation we can postulate that it is the formation of these chains versus that of discrete dimers related by rotation or pseudorotation observed where *Z*ˈ > 1 that determines the high *Z*ˈ character of the structure. Furthermore, it seems likely that where a molecule possesses the specific stereochemistry of the cyclohexane moiety of a shikimate and in the absence of any other hydrogen bond donors on the molecule, the structure has a high probability of exhibiting a high *Z*ˈ value.

With the most likely factors that dictate high *Z*ˈ values in shikimates now identified, it is worth considering how these crystal structures compare to other known high *Z*ˈ structures. In their comprehensive meta-analysis of high *Z*ˈ structures, Brock identified the structural features associated with very high *Z*ˈ values (*Z*ˈ > 4) and documented the frequency of these features in a large number of very high *Z*ˈ structures from the CSD.^20^ As might be expected, a number of these features can be observed in the structures reported herein where *Z*ˈˈ > 1. The occurrence of these features in **1**, **2**, **4**, and VUXROV is illustrated in Table 1.

**Table 1:** Presence of Various Features Associated with the Packing of Very High *Z*ˈ Structures in **1**, **2**, **4**, and VUXROV

	**1**	**2**	**4**	VUXROV
*Z*ˈ/*Z*ˈˈ	12/12	2/2	1/3	2/2
*Z*ˈ is even	yes	yes	no	yes
negligible conformational variation	yes	yes	yes	yes
strong intermolecular interactions	yes	yes	yes	yes
translational modulations	yes	no	no	no
pseudosymmetry	yes	yes	no	no
Sohncke space group	yes	yes	yes	yes
layered structure	yes	yes	yes	no
multiple features (*Z*ˈ × 2 or 3)	yes	no	no	no
other (order faults, etc.)	yes	no	no	Nn

It can be seen that the structure of **1** possesses all of the features Brock identified as being associated with a very high *Z*ˈ, which is not surprising given its unusually high *Z*ˈ value. Though several of these factors can only apply to structures with very high *Z*ˈ values (e.g., the multiple features that can double or triple the value of *Z*ˈ) a number are observed in all four of the structures in the table including **4** which, as a cocrystal, has been included on the basis of its *Z*ˈˈ value. It can hence be said that the high *Z*ˈ crystal structures that comprise this work compare favorably to those already known. That so many of the most common features in very high *Z*ˈ structures were observed in **1** is surely a sign that these factors and their frequencies could be put to use in crystal structure prediction so that possible high *Z*ˈ structures are not overlooked.

With a view to building on the analysis of these high *Z*ˈ structures, the rational design of molecules with similar properties that would also form structures with a high *Z*ˈ was perhaps a logical next step. In the development of a suitable compound, the decision was made to start with the trihydroxycyclohexene core of the shikimate compounds described previously to retain the conformational inflexibility and chirality that gave them a high probability of producing high *Z*ˈ crystal structures. In this case, however, the substituent on the 1-position of this ring was to be an amide.

Comparing the acid and ester structures, it was clear that the size of this substituent and its ability to form classical hydrogen bonds (or rather, inability) is a key factor in determining the *Z*ˈ value of the crystal structure and hence an amide substituent with no hydrogen bond donors and of a smaller size than an *iso*-propoxy group was determined to give the greatest probability of producing a structure with *Z*ˈ> 1. As a dimethylamino group matches these criteria, it was concluded that it would be suitable for this purpose.

As predicted, the structure of the dimethylamino shikimate amide (**6**) crystallized with *Z*ˈ > 1 with a *Z*ˈ value of 2. In contrast to the structures described previously, the structure of **6** was observed to be a chloroform solvate with one solvent molecule for each molecule of amide, giving a *Z*ˈˈ value of 4 for the asymmetric unit. Each chloroform molecule forms a weak C–H···O hydrogen bond with the carbonyl oxygen atom of the amide group.

Superficially, this structure possesses many of the same features observed in the other homomolecular shikimate structures where *Z*ˈ> 1. The asymmetric unit appears to be a hydrogen-bonded R^2^_2_(10) dimer, and the complete hydrogen bonding network forms two-dimensional sheets, in this case coplanar with the crystallographic (001) plane. However, a more thorough analysis of the hydrogen bonding in this structure reveals that these features form in a very different way to those of the aforementioned structures. First, instead of comprising discrete R^2^_2_(10) dimer units formed by interactions between the hydroxyl groups containing the O1 and O2 atoms as in **1** and **2**, in the case of **6** each molecule forms two R^2^_2_(10) ring motifs to neighboring molecules via O1–H···O2 and O2–H···O3 interactions. This is similar to the hydrogen bonding network observed in **3** that also forms chains of R^2^_2_(10) rings through interactions involving the same hydroxyl groups. In **3** each molecule in the chain was related by symmetry giving a structure with *Z*ˈ = 1 but this cannot be the case for **6**.

**Figure 7:**
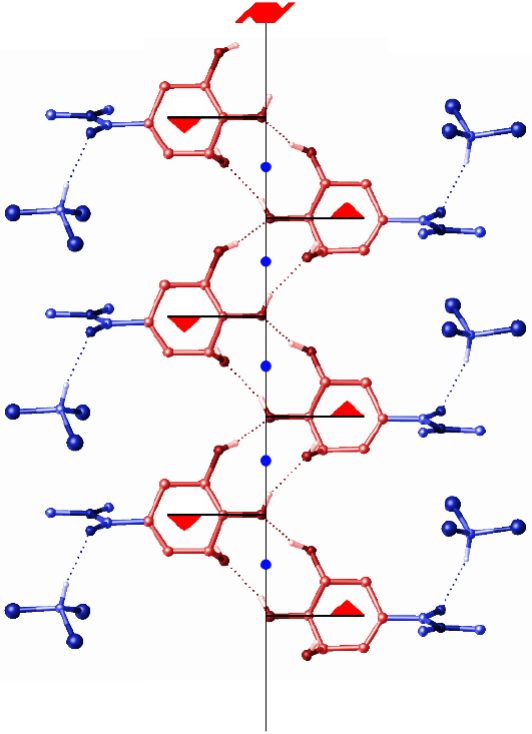
A view of the hydrogen-bonded chain of rings in **6** highlighting the different symmetry relationships between two different parts of the structure. The cyclohexyl rings (red) are related by a 2_1_ screw axis, whereas the amide groups and chloroform molecules (blue) are related by inversion.

On closer inspection, it is not immediately obvious as to why the two molecules are not related by symmetry. Considering the neighboring molecules in the chain of hydrogen-bonded rings, there appears to be little difference in the conformation of the shikimate moiety (both the cyclohexene ring and the hydroxyl groups) of each successive molecule, and these moieties are clearly related by a 2_1_ screw along the [010] direction as in the structure of **3**. However, examining the relationship between the amide groups on neighboring molecules in the chain reveals how the rotational symmetry is broken. Where the cyclohexene and hydroxyl groups are related by pseudoscrew symmetry, the amide groups appear to be related by a pseudoinversion (Figure 7). This is in part due to the unusual orientation of the amide group compared to the ester and acid groups in the **1**–**5**. Where in these structures the C6–C1–C7–O4 torsion angle did not exceed 30°, the equivalent angles in **6** are 76.8(5)° and 115.4(4)°. These larger torsions provide space for the chloroform molecules in the structure and, as these molecules are also related by pseudoinversion, likely play a role in breaking the symmetry which leads to the *Z*ˈ value of 2.

The orientations of the two molecules within the chain are also significantly different in **6** relative to **3**. Where in **3** the chain was formed in a herringbone arrangement, the molecules in the hydrogen-bonded chain in **6** are orientated parallel to each other in a stepwise fashion. The most obvious change in the intermolecular geometry that results from this is seen in the direction of one of the hydrogen bonds, as described by the H–C3–O1–H torsion angle, which is ca. 170° in the structure of **3** but only ca. 31° in that of **6**. The stepwise orientation is also favored by both the small steric effect of the dimethylamide group relative to the *iso*-propyl ester group and the presence of the solvent interacting with the carbonyl oxygen of the amide group in **6**.

Another structural difference between **6** and **1**–**3** can be observed in the way the chains of the rings link together to form 2D sheets of hydrogen-bonded molecules. Sheet motifs of this kind are common to all of these structures but in those of the shikimate esters, only the hydroxyl groups are involved in their formation. In **6**, the carbonyl oxygen atoms of the amide groups act as hydrogen bond acceptors, and it is these interactions that form the links between dimers and propagate the hydrogen bonding network in the second dimension. There are no hydrogen bonds observed to the carbonyl oxygen atom in any of the homomolecular ester structures reported in this work. These hydrogen bonds are clearly another reason behind the unusual orientation of the amide group relative to the cyclohexene ring in this structure.

The hydrogen bond accepted by the amide group and the lack of any bifurcated hydrogen bonds in the structure result in there being one hydroxyl oxygen atom that does not act as a hydrogen bond acceptor. This is similar to one of the hydrogen bonding environments in the structure of **1** where weak C–H···O interactions were observed. Near-identical interactions in terms of both the atoms involved and the interatomic distances are also observed in the case of **6** completing the hydrogen bonding at about the point of O1 and O4.

Bearing all of this analysis in mind, though **6** does have a high *Z*ˈ value, it appears that it is the presence of the solvent in the structure that causes this. Were it not present, the C6–C1–C7–O4 torsion angle might be closer to those observed in the esters and the hydrogen bonding network might be closer that of the structure of **3**. Considering the packing in the structure it is not simply the presence of the solvent that gives two independent molecules but more importantly the symmetry relationship between them. By inspection of the packing, it would seem that the solvent could theoretically have packed in such a way as to be related by the screw axis, as the trihydroxycyclohexene rings are, without the need for any rearrangement of the rest of the molecule. Despite the possible influence of the solvent, it is still encouraging that, as predicted, the structure exhibits *Z*ˈ> 1.

## Conclusions

In addition to addressing the lack of structurally characterized shikimates, this work identifies the likely roots of the large *Z*ˈ values observed for these structures and postulates that, due to their awkward shape, chirality, and asymmetry, shikimates are more likely than not to crystallize with high *Z*ˈ values (or high *Z*ˈˈ values in the case of cocrystals). Though there is only so much one can conclude from a study of such a small sample size, the shikimate ester structures determined as part of this work along with the sole structurally characterized shikimate in the literature seem to suggest that molecules of this type are prone to high *Z*ˈ values.

Though the exact way in which the symmetry-independent molecules interact to form the asymmetric units of the high *Z*ˈ structures reported here is different in each case, the molecular characteristics that allow for high *Z*ˈ values are the same. Considering the shikimate esters alone, it can also be concluded that, due to increasing steric bulk and the unfavorable intermolecular interactions associated with this, the larger the ester group of a given shikimate ester, the lower the value of *Z*ˈ. In each case, the ester group acts to prevent the propagation of the hydrogen bonding in certain directions, leading to frustration between the packing of the asymmetric units and the intermolecular symmetry.

Where all the structures analyzed in this study are considered, a high *Z*ˈ value is always observed where the hydrogen-bonded R^2^_2_(10) dimer motif involving the hydroxyl groups containing the O1 and O2 atoms of two adjacent molecules is formed as opposed to extended chains of hydrogen-bonded molecules related by symmetry operations with translational components. It may therefore be possible to engineer specific molecules which favor the formation of these dimers and produce further structures with high *Z*ˈ values. Furthermore, the insights provided by this study may inform the study of more diverse structures where *Z*ˈ> 1 specifically regarding their polymorphism and crystal structure prediction.
