# Supplementary material for: A State of Independents: Rationalizing the High Zˈ Crystal Structures of Shikimate Esters
Source: Cryst Growth Des. 2024 Jan 16;24(3):1429–37. doi: 10.1021/acs.cgd.3c01383 (PMC10853928; doi:10.1021/acs.cgd.3c01383)
Supplement: Supplementary file 1 — cg3c01383_si_001.pdf [file cg3c01383_si_001.pdf]

Supporting Information

A state of independents: rationalizing the high Z' structures of shikimate esters

Ronnie Ragbirsingh, Michael J. Hall, Michael R. Probert, Paul G. Waddell

Chemistry, School of Natural and Environmental Sciences, Newcastle University, Bedson Building, Newcastle upon Tyne NE1 7RU, UK

Corresponding author e-mail: paul.waddell@ncl.ac.uk

Appendix A: Single Crystal X-ray Crystallography Data

Methyl (3*R*,4*S*,5*R*)-3,4,5-trihydroxycyclohex-1-ene-1-carboxylate (1)

Table S1: Crystal structure and refinement data for **1**

Empirical formula	$C_8H_{12}O_5$
Formula weight	188.18
Temperature/K	100.0(2)
Crystal system	monoclinic
Space group	$P2_1$
a/Å	25.5964(6)
b/Å	6.38050(10)
c/Å	33.7807(7)
α/°	90
β/°	108.702(2)
γ/°	90
Volume/Å^3	5225.69(19)
Z	24
ρ_{calc}g/cm^3	1.435
μ/mm^{-1}	0.113
F(000)	2400.0
Crystal size/mm^3	0.331 × 0.022 × 0.01
Radiation	Synchrotron (λ = 0.6889 Å)
2Θ range for data collection/°	1.628 to 53.146
Index ranges	-33 ≤ h ≤ 33, -8 ≤ k ≤ 8, -43 ≤ l ≤ 43
Reflections collected	73705
Independent reflections	23672 [R_{int} = 0.0887, R_{sigma} = 0.0870]
Data/restraints/parameters	23672/37/1528
Goodness-of-fit on F^2	0.925
Final R indexes [I>=2σ (I)]	R_1 = 0.0498, wR_2 = 0.1198
Final R indexes [all data]	R_1 = 0.0865, wR_2 = 0.1339
Largest diff. peak/hole / e Å^{-3}	0.33/-0.27
Flack parameter	1.9(4)

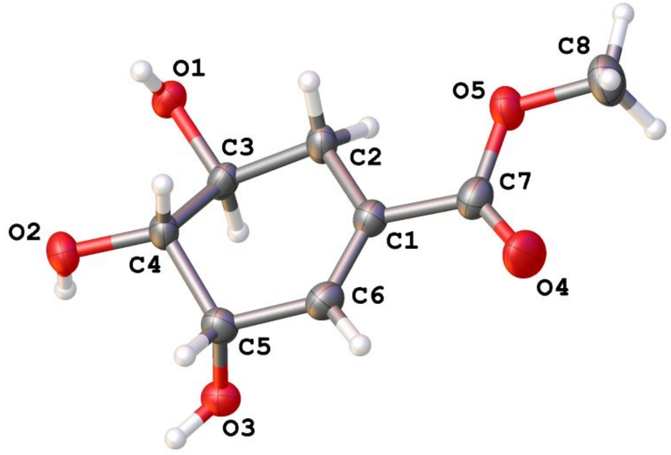

Figure S1: A representative molecule from the asymmetric unit of **1** with atomic displacement parameters (ADPs) drawn to the 50% probability level.

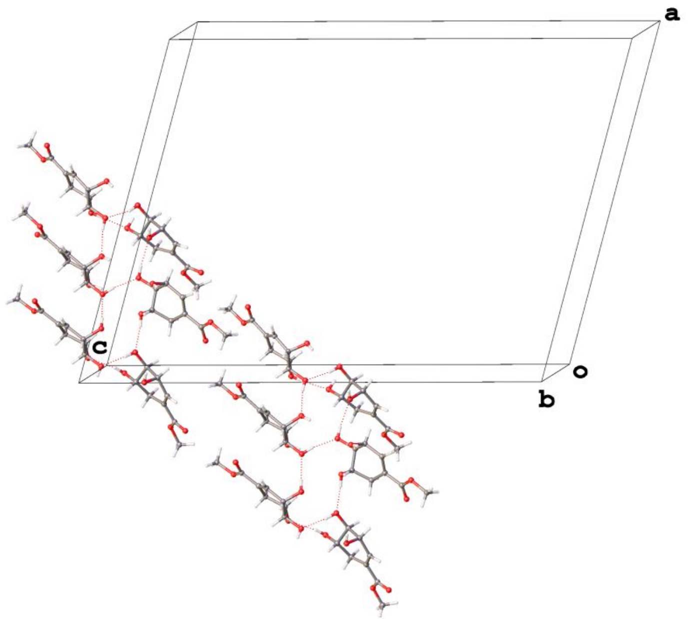

Figure S2: Asymmetric unit of **1** with hydrogen atoms bound to carbon omitted for clarity. Hydrogen bonds are indicated by dashed lines.

Ethyl (3*R*,4*S*,5*R*)-3,4,5-trihydroxycyclohex-1-ene-1-carboxylate (2)

Table S2: Crystal structure and refinement data for **2**

Empirical formula	$C_9H_{14}O_5$
Formula weight	202.20
Temperature/K	150.0(2)
Crystal system	monoclinic
Space group	C2
a/Å	16.7981(6)
b/Å	6.43158(19)
c/Å	19.1864(8)
α/°	90
β/°	106.640(4)
γ/°	90
Volume/Å^3	1986.06(13)
Z	8
ρ_{calc}g/cm^3	1.352
μ/mm^{-1}	0.942
F(000)	864.0
Crystal size/mm^3	0.29 × 0.05 × 0.02
Radiation	CuKα (λ = 1.54184 Å)
2Θ range for data collection/°	9.622 to 133.824
Index ranges	$-20 \leq h \leq 19$, $-6 \leq k \leq 7$, $-22 \leq l \leq 22$
Reflections collected	7668
Independent reflections	3104 [R_{int} = 0.0412, R_{sigma} = 0.0467]
Data/restraints/parameters	3104/462/347
Goodness-of-fit on F^2	1.045
Final R indexes [I>=2σ (I)]	R_1 = 0.0386, wR_2 = 0.0880
Final R indexes [all data]	R_1 = 0.0499, wR_2 = 0.0954
Largest diff. peak/hole / e Å^{-3}	0.18/-0.16
Flack parameter	-0.01(16)

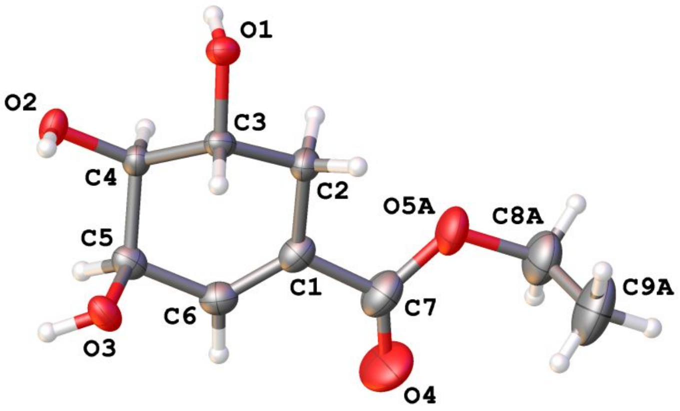

Figure S3: A representative molecule from the asymmetric unit of **2** with ADPs drawn at the 50% probability level. As ethyl group in this structure has been modelled as disordered over two positions only the site with major occupancy has been shown.

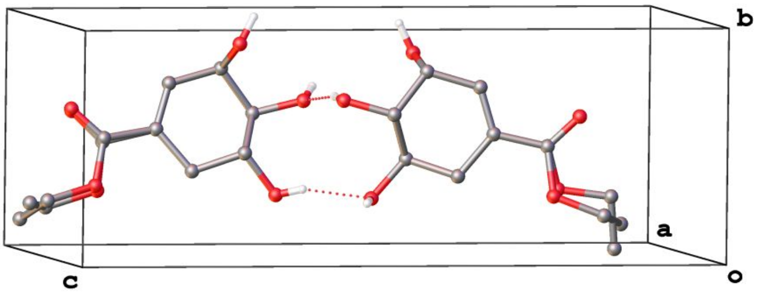

Figure S4: The asymmetric unit of **2** with hydrogen atoms bound to carbon omitted for clarity. Hydrogen bonds are indicated by dashed lines.

Isopropyl (3*R*,4*S*,5*R*)-3,4,5-trihydroxycyclohex-1-ene-1-carboxylate (3)

Table S3: Crystal structure and refinement data for **3**

Empirical formula	$C_{10}H_{16}O_5$
Formula weight	216.23
Temperature/K	150.0(2)
Crystal system	monoclinic
Space group	$P2_1$
a/Å	5.8032(4)
b/Å	7.5952(5)
c/Å	12.0400(11)
α/°	90
β/°	90.067(7)
γ/°	90
Volume/Å^3	530.68(7)
Z	2
ρ_{calc}g/cm^3	1.353
μ/mm^{-1}	0.916
F(000)	232.0
Crystal size/mm^3	0.33 × 0.04 × 0.02
Radiation	CuKα (λ = 1.54184)
2Θ range for data collection/°	7.342 to 133.748
Index ranges	$-6 \leq h \leq 6$, $-9 \leq k \leq 8$, $-14 \leq l \leq 14$
Reflections collected	7597
Independent reflections	1692 [R_{int} = 0.0541, R_{sigma} = 0.0416]
Data/restraints/parameters	1692/112/149
Goodness-of-fit on F^2	1.059
Final R indexes [I>=2σ (I)]	R_1 = 0.0361, wR_2 = 0.0853
Final R indexes [all data]	R_1 = 0.0392, wR_2 = 0.0882
Largest diff. peak/hole / e Å^{-3}	0.18/-0.17
Flack parameter	-0.03(18)

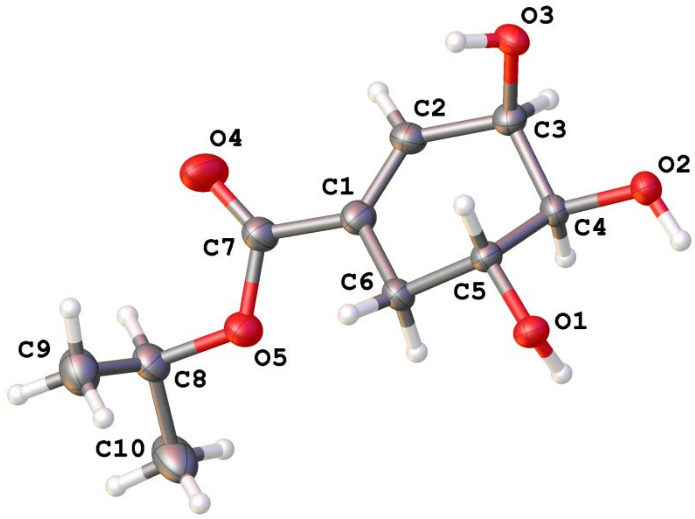

Figure S5: The asymmetric unit of **3** with ADPs drawn at the 50% probability level.

Shikimic acid, propyl (3*R*,4*S*,5*R*)-3,4,5-trihydroxycyclohex-1-ene-1-carboxylate 2:1 co-crystal (4)

Table S4: Crystal structure and refinement data for **4**

Empirical formula	$C_{24}H_{36}O_{15}$
Formula weight	564.53
Temperature/K	150.0(2)
Crystal system	monoclinic
Space group	$P2_1$
a/Å	5.86051(13)
b/Å	7.83710(18)
c/Å	27.3953(8)
α/°	90
β/°	89.357(2)
γ/°	90
Volume/Å^3	1258.17(6)
Z	2
ρ_{calc}g/cm^3	1.490
μ/mm^{-1}	1.072
F(000)	600.0
Crystal size/mm^3	0.25 × 0.18 × 0.02
Radiation	CuKα (λ = 1.54184 Å)
2Θ range for data collection/°	9.686 to 133.866
Index ranges	$-6 \le h \le 6$, $-9 \le k \le 9$, $-32 \le l \le 31$
Reflections collected	17478
Independent reflections	4438 [R_{int} = 0.0484, R_{sigma} = 0.0411]
Data/restraints/parameters	4438/1/387
Goodness-of-fit on F^2	1.050
Final R indexes [I>=2σ (I)]	R_1 = 0.0347, wR_2 = 0.0800
Final R indexes [all data]	R_1 = 0.0411, wR_2 = 0.0837
Largest diff. peak/hole / e Å^{-3}	0.18/-0.17
Flack parameter	-0.03(11)

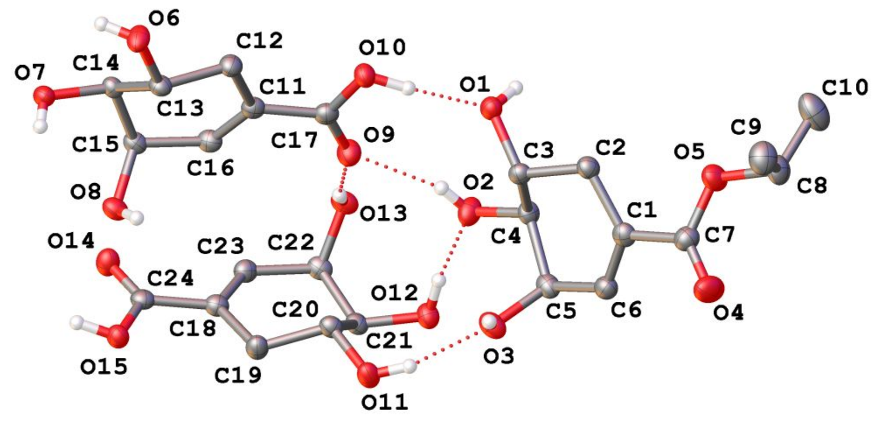

Figure S6: The asymmetric unit of **4** with ADPs drawn at the 50% probability level and hydrogen atoms bound to carbon omitted for clarity. Hydrogen bonds are indicated by dashed lines.

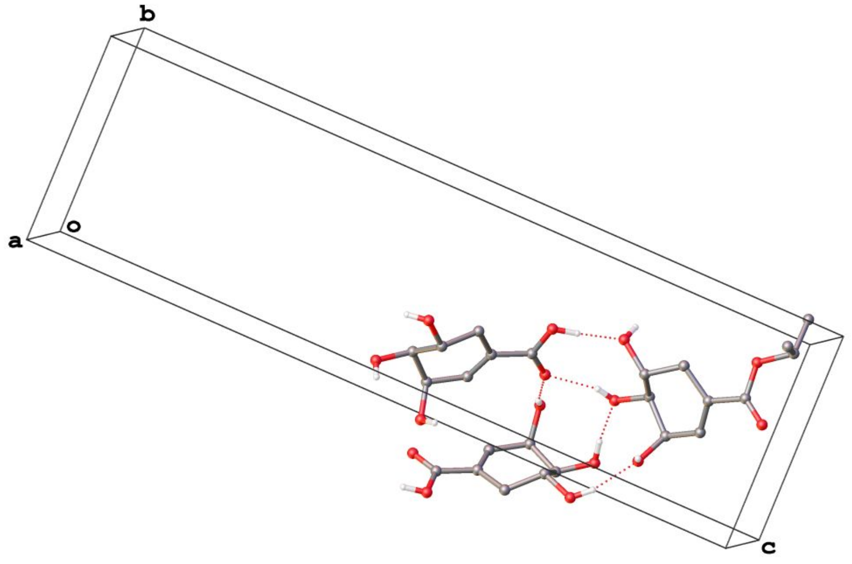

Figure S7: The asymmetric unit of **4** relative to the unit cell axes with hydrogen atoms bound to carbon omitted for clarity. Hydrogen bonds are indicated by dashed lines.

Shikimic acid (5)

Table S5: Crystal structure and refinement data for **5**

Empirical formula	$C_7H_{10}O_5$
Formula weight	174.15
Temperature/K	150.0(2)
Crystal system	orthorhombic
Space group	$P2_12_12_1$
a/Å	5.81401(15)
b/Å	7.96608(17)
c/Å	15.8167(5)
α/°	90
β/°	90
γ/°	90
Volume/Å^3	732.55(3)
Z	4
ρ_{calc}g/cm^3	1.579
μ/mm^{-1}	1.177
F(000)	368.0
Crystal size/mm^3	0.36 × 0.07 × 0.01
Radiation	CuKα (λ = 1.54184 Å)
2Θ range for data collection/°	11.188 to 133.338
Index ranges	$-5 \leq h \leq 6$, $-9 \leq k \leq 9$, $-18 \leq l \leq 18$
Reflections collected	10361
Independent reflections	1297 [R_{int} = 0.0612, R_{sigma} = 0.0313]
Data/restraints/parameters	1297/0/121
Goodness-of-fit on F^2	1.078
Final R indexes [I>=2σ (I)]	R_1 = 0.0303, wR_2 = 0.0729
Final R indexes [all data]	R_1 = 0.0346, wR_2 = 0.0757
Largest diff. peak/hole / e Å^{-3}	0.15/-0.16
Flack parameter	0.03(15)

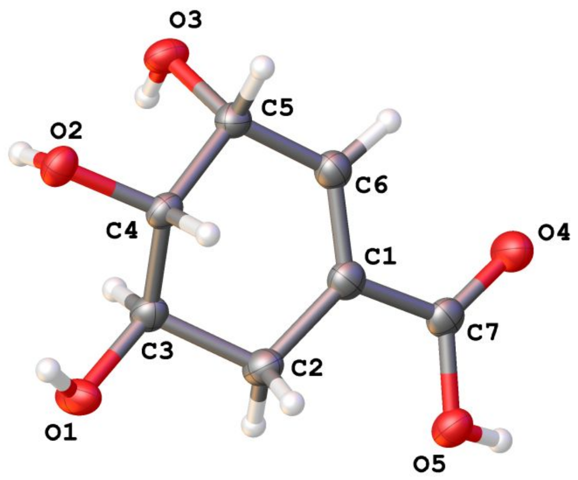

Figure S8: The asymmetric unit of **5** with ADPs drawn at the 50% probability level.

Dimethyl (3*R*,4*S*,5*R*)-3,4,5-trihydroxycyclohex-1-ene-1-amide chloroform solvate (6)

Table S6: Crystal structure and refinement data for **6**

Empirical formula	$C_{10}H_{16}Cl_3NO_4$
Formula weight	320.59
Temperature/K	150.0(2)
Crystal system	triclinic
Space group	P1
a/Å	6.0239(2)
b/Å	7.3958(2)
c/Å	16.0305(6)
α/°	97.738(3)
β/°	93.627(3)
γ/°	93.971(3)
Volume/Å^3	704.07(4)
Z	2
ρ_{calc}g/cm^3	1.512
μ/mm^{-1}	5.971
F(000)	332.0
Crystal size/mm^3	0.33 × 0.13 × 0.02
Radiation	CuKα (λ = 1.54184)
2Θ range for data collection/°	11.17 to 133.652
Index ranges	-7 ≤ h ≤ 6, -8 ≤ k ≤ 8, -19 ≤ l ≤ 19
Reflections collected	19148
Independent reflections	4816 [R_{int} = 0.0383, R_{sigma} = 0.0320]
Data/restraints/parameters	4816/3/347
Goodness-of-fit on F^2	1.048
Final R indexes [I>=2σ (I)]	R_1 = 0.0293, wR_2 = 0.0694
Final R indexes [all data]	R_1 = 0.0345, wR_2 = 0.0726
Largest diff. peak/hole / e Å^{-3}	0.21/-0.19
Flack parameter	-0.010(11)

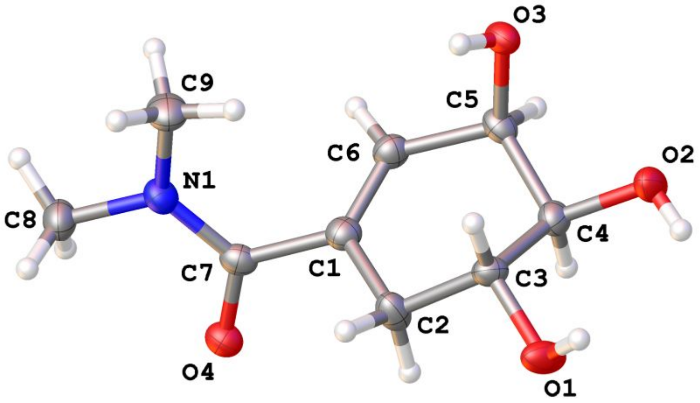

Figure S9: A representative molecule from the asymmetric unit of **6** with ADPs drawn at the 50% probability level.

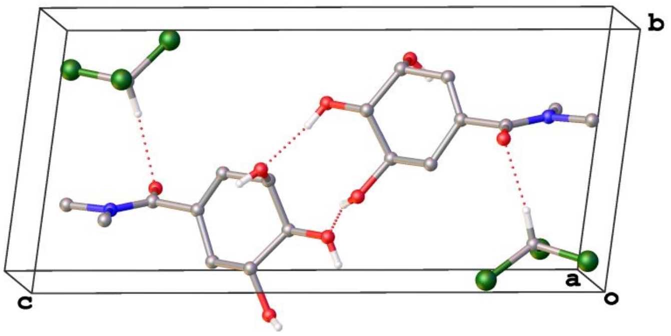

Figure S10: The asymmetric unit of **6** relative to the unit cell axes with hydrogen atoms bound to carbon omitted for clarity. Hydrogen bonds are indicated by dashed lines.

Appendix B: Synthesis and Characterisation

Methyl (3*R*,4*S*,5*R*)-3,4,5-trihydroxycyclohex-1-ene-1-carboxylate (1)

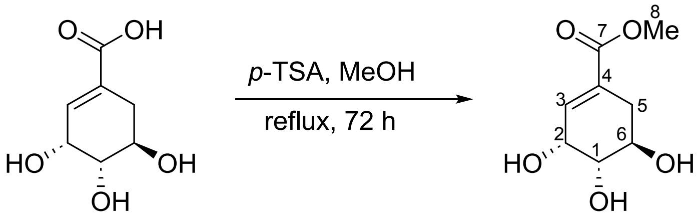

To a stirred round bottom flask (250 mL) was added shikimic acid (5.00 g, 28.7 mmol), MeOH (125 mL) and *p*-toluene sulfonic acid monohydrate (0.50 g, 2.6 mmol). The resulting solution was put under a nitrogen atmosphere, heated to reflux and the reaction was monitored by TLC. After 72 h the reaction mixture was cooled to rt, filtered and the solvent removed under reduced pressure. The crude product was recrystallized from hot EtOAc (15 mL) to afford methyl (3*R*,4*S*,5*R*)-3,4,5-trihydroxycyclohex-1-ene-1-carboxylate as an off-white crystalline solid (3.97 g, 21 mmol, 73 %).

^{1}H NMR (300 MHz, MeOD-d_4) δ_H 6.79 (1H, dtd, J = 3.7, 1.9, 0.7 Hz, H^3), 4.40 – 4.34 (1H, m, H^6), 4.01 (1H, dddd, J = 7.1, 5.4, 4.9, 0.6 Hz, H^1), 3.74 (3H, s, H^8), 3.69 (1H, dd, J = 7.2, 4.1 Hz, H^2), 2.76 – 2.62 (1H, m, H^5), 2.20 (1H, ddt, J = 18.2, 5.4, 1.7 Hz, H^5); ^{13}C NMR (75 MHz, MeOD-*d*4) δ_C 167.3, 137.7, 128.8, 71.1, 67.0, 65.8, 50.9, 30.1; IR (neat): v_{max}cm^{-1} 3311 (OH), 1717 (s, C=O).

Ethyl (3*R*,4*S*,5*R*)-3,4,5-trihydroxycyclohex-1-ene-1-carboxylate (2) S2

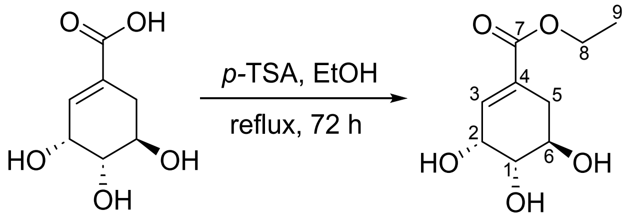

To a stirred round bottomed flask (250 mL) was added shikimic acid (5.00 g, 28.7 mmol), EtOH (125 mL) and *p*-toluene sulfonic acid monohydrate (0.50 g, 2.6 mmol). The resulting solution was put under a nitrogen atmosphere, heated to reflux and monitored by TLC. After 72 h the reaction mixture was cooled to rt, filtered and the solvent removed under reduced pressure. The crude product was recrystallized from hot EtOAc (15 mL) to afford ethyl (3*R*,4*S*,5*R*)-3,4,5-trihydroxycyclohex-1-ene-1-carboxylate as an off-white crystalline solid (4.35 g, 22 mmol, 75%).

^{1}H NMR (300 MHz, Chloroform-*d*) δ 6.91 – 6.88 (1H, m, H^3), 4.47 (1H, t, J = 4.6 Hz, H^2), 4.22 (2H, q, J = 7.1 Hz, H^8), 3.98 (1H, td, J = 9.5, 5.5 Hz, H^6), 3.63 (1H, dd, J = 9.6, 4.6 Hz, H^1), 2.96 (1H, dd, J = 17.9, 5.5 Hz, H^5), 2.28 – 2.16 (1H, m, H^5), 1.30 (3H, t, J = 7.1 Hz, H^9); ^{13}C NMR (75 MHz, Chloroform-*d*) δ_C 166.1, 135.0, 131.5, 73.1, 67.0, 66.0, 61.1, 32.1, 14.1; IR (neat): v_{max}cm^{-1} 3330 (OH), 1713 (C=O).

Isopropyl (3*R*,4*S*,5*R*)-3,4,5-trihydroxycyclohex-1-ene-1-carboxylate (3)

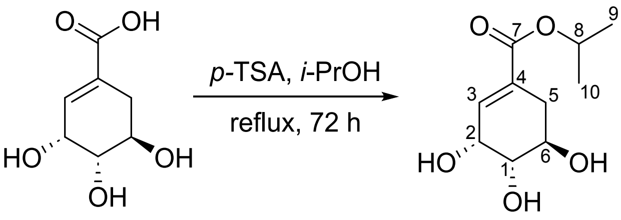

To a stirred round bottomed flask (250 mL) was added shikimic acid (5.00 g, 28.7 mmol), followed by *i*-PrOH (125 mL) and then *p*-toluene sulfonic acid monohydrate (0.50 g, 2.6 mmol). The resulting solution was put under a nitrogen atmosphere, heated to reflux and monitored by TLC. After 72 h the reaction mixture was cooled to rt, filtered and the solvent removed under reduced pressure. The crude product was purified by column chromatography (DCM: MeOH, 9: 1) to afford isopropyl (3*R*,4*S*,5*R*)-3,4,5-trihydroxycyclohex-1-ene-1-carboxylate as a colourless crystalline solid (4.59 g, 21 mmol, 74 %).

^{1}H NMR (300 MHz, Chloroform-*d*) δ 6.86 (1H, ddd, J = 4.9, 2.6, 1.1 Hz, H^3), 5.07 (1H, hept, J = 6.2 Hz, H^8), 4.46 (1H, t, J = 4.7 Hz, H^2), 3.97 (1H, td, J = 9.0, 5.6 Hz, H^6), 3.62 (1H, dd, J = 9.5, 4.6 Hz, H^1), 2.94 (1H, dd, J = 17.9, 5.6 Hz, H^5), 2.33 (3H, br s, 3xOH), 2.26 – 2.15 (1H, m, H^5), 1.27 (6H, d, J = 6.2 Hz, $H^{9 \text{ and } 10}$); ^{13}C NMR (75 MHz, Chloroform-*d*) δ_C 165.7, 134.8, 132.0, 73.3, 68.7, 67.2, 66.1, 32.3, 21.9; IR (neat): v_{max}cm^{-1} 3313 (OH), 1714 (C=O).

(3*R*,4*S*,5*R*)-3,4,5-trihydroxy-*N*,*N*-dimethylcyclohex-1-ene-1-carboxamide (6)

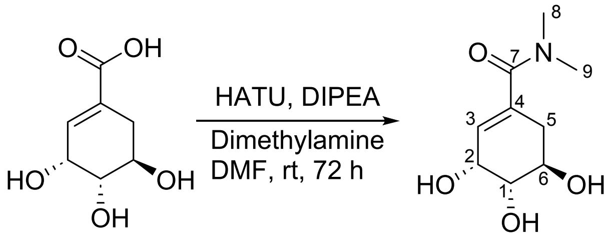

To a stirred round bottomed flask (250 mL) under a nitrogen atmosphere was added shikimic acid (1.00 g, 5.7 mmol), followed by HATU (2.6 g, 6.9 mmol). Dry DMF (25 mL) was then added by syringe, the reaction mixture stirred for 5 min, followed by the addition of DIPEA (1.5 mL, 8.6 mmol) and then *N*,*N*-dimethylamine (0.97 mL, 8.6 mmol). The resultant reaction mixture was stirred at rt for 72 h, after which the solvent was removed under reduced pressure and the crude reaction material purified by column chromatography (DCM/MeOH, 4: 1) to give (3*R*,4*S*,5*R*)-3,4,5-trihydroxy-*N*,*N*-dimethylcyclohex-1-ene-1-carboxamide as a clear colourless oil (0.39 g, 1.9 mmol, 34 %).

^{1}H NMR (300 MHz, $CDCl_3$) δ 5.82 (1H, s, H^3), 4.77 (1H, s, OH), 4.70 (2H, s, 2x OH), 4.38 (1H, br s, H^2), 4.06 – 3.98 (1H, m, H^6), 3.68 – 3.60 (1H, m, H^1), 3.03 (3H, s, $H^{8 \text{ or } 9}$), 2.97 (3H, s, $H^{8 \text{ or } 9}$), 2.69 (1H, dd, J = 17.5, 5.4 Hz, H^{5a}), 2.27 (1H, dd, J = 17.3, 7.7 Hz, H^{5b}); ^{13}C NMR (75 MHz, $CDCl_3$) δ 171.6, 135.2, 126.7, 73.1, 66.6, 66.6, 38.9, 35.0, 33.7; IR (neat): v_{max}cm^{-1} 3303 (OH), 1603 (C=O).

Supplemental References

S1 L. Chahoua, M. Baltas, L. Gorrichon, P. Tisnes and C. Zedde, *The Journal of Organic Chemistry*, 1992, **57**, 5798–5801. https://doi.org/10.1021/jo00047a049

S2 Y. Yamashita, K. Hanaya, T. Sugai, T. Mizushima and M. Shoji, *Tetrahedron*, 2013, **69**, 6527–6532. https://doi.org/10.1016/j.tet.2013.05.004
